# Transcriptome Analysis of High-NUE (T29) and Low-NUE (T13) Genotypes Identified Different Responsive Patterns Involved in Nitrogen Stress in Ramie (*Boehmeria nivea* (L.) Gaudich)

**DOI:** 10.3390/plants9060767

**Published:** 2020-06-19

**Authors:** Longtao Tan, Gang Gao, Chunming Yu, Aiguo Zhu, Ping Chen, Kunmei Chen, Jikang Chen, Heping Xiong

**Affiliations:** 1 Institute of Bast Fiber Crops, Chinese Academy of Agricultural Sciences, Changsha 410205, China; tanlongtao@isa.ac.cn (L.T.); gaogang@caas.cn (G.G.); yuchunming@caas.cn (C.Y.); zhuaiguo@caas.cn (A.Z.); Chenping02@caas.cn (P.C.); Chenkunmei@caas.cn (K.C.); 2 Institute of Subtropical Agriculture, The Chinese Academy of Sciences, Changsha 410125, China

**Keywords:** ramie (*Boehmeria nivea* (L.) Gaudich), transcriptome, nitrogen deficiency, resilience, nitrogen-use efficiency

## Abstract

Nitrogen-use efficiency (NUE) has significant impacts on plant growth and development. NUE in plants differs substantially in physiological resilience to nitrogen stress; however, the molecular mechanisms underlying enhanced resilience of high-NUE plants to nitrogen deficiency remains unclear. We compared transcriptome-wide gene expression between high-NUE and low-NUE ramie (*Boehmeria nivea* (L.) Gaudich) genotypes under nitrogen (N)-deficient and normal conditions to identify the transcriptomic expression patterns that contribute to ramie resilience to nitrogen deficiency. Two ramie genotypes with contrasting NUE were used in the study, including T29 (NUE = 46.01%) and T13 (NUE = 15.81%). Our results showed that high-NUE genotypes had higher gene expression under the control condition across 94 genes, including frontloaded genes such as GDSL esterase and lipase, gibberellin, UDP-glycosyltransferase, and omega-6 fatty acid desaturase. Seventeen stress-tolerance genes showed lower expression levels and varied little in response to N-deficiency stress in high-NUE genotypes. In contrast, 170 genes were upregulated under N deficiency in high-NUE genotypes but downregulated in low-NUE genotypes compared with the controls. Furthermore, we identified the potential key genes that enable ramie to maintain physiological resilience under N-deficiency stress, and categorized these genes into three groups based on the transcriptome and their expression patterns. The transcriptomic and clustering analysis of these nitrogen-utilization-related genes could provide insight to better understand the mechanism of linking among the three gene classes that enhance resilience in high-NUE ramie genotypes.

## 1. Introduction

Increasing population and consumption are placing unprecedented demands on agriculture and natural resources [1]. One of the greatest challenges of the 21st century is meeting the world’s growing need for agricultural products while simultaneously reducing agriculture’s environmental harm. Improvements in nitrogen-use efficiency (NUE) in crop production are critical for addressing the challenge [2]. Therefore, the development and cultivation of new varieties containing genetic traits associated with abiotic stress tolerance will be essential in order to sustainably grow high-yielding crops under increasingly stressful environmental conditions [3].

Fiber extracted from ramie (*Boehmeria nivea* L.) stem is the longest and one of the strongest natural fine textile fibers [4]. In recent years, the rapidly growing application of ramie for addressing needs of feed [5,6], phytoremediation [7], and mushroom production [8] has made the crop a research hotspot, and the commercial cultivation of this crop has increased in countries such as China, Brazil, and the Philippines [9]. Nitrogen is the primary nutrient required for optimal growth and fiber yield [10,11,12]. Despite the agronomic importance of ramie, our previous studies showed that nitrogen fertilizer was applied excessively in ramie fields and ramie presented a very low nitrogen agronomy efficiency (NAE, 23.2–27.8%) in traditional farming systems. In addition, ramie was mainly planted in Yangtze River Valley in China, which exhibits a rapid alternation from droughts to floods. Hence, decreasing the human and environmental costs and risks associated with nitrogen loss and pollution in ramie fields is critical.

It is imperative to understand the molecular mechanisms of stress tolerance for breeding and cultivation of high-NUE genotypes [13,14]. Some genetic variability in N uptake has been thoroughly investigated [15,16] and genome-wide responses to low-nitrogen (N) stress have been described in many plants [17,18,19,20,21]. There have also been a few studies discussing N utilization and metabolism in ramie [22], but none considered N-stress simultaneously with genotypes. Previous studies mainly focused on differentially expressed genes (DEGs) that are more sensitive to nitrogen rates and had higher plasticity during genetic modification, especially those genes expressed much more highly in high-NUE genotypes than in low-NUE genotypes. However, without comparing the transcriptional differences between low-N-tolerant and low-N-sensitive genotypes, separating stress-tolerance genes from stress-responsive genes was impossible [23]. Additionally, the express pattern of constitutive primed genes in higher-tolerance genotypes cannot be detected by a single type of experiment due to the variety specificity. Therefore, we hypothesized that classification of express patterns of ramie DEGs by testing genotypes with contrasting NUE would increase our understanding of the N-utilization mechanism and enable us to focus on targeted genes.

In the present study, we used *de novo* transcriptome assembly and digital gene expression (DGE) profiling to measure gene expression differences between high- and low-NUE genotypes under nitrogen-deficient (0 mmol·L^−1^) and normal conditions (10 mmol·L^−1^). The objectives were to investigate the effects of N deficiency on the gene expression of two contrasting ramie varieties differing in NUE, and the functional classification of DEGs was conducted to explore the resilience mechanism of ramie.

## 2. Materials and Methods

### 2.1. Plant Material and N Treatment

Two ramie varieties, H2000-03 (T29) and Ceheng Jiama (T13), were identified to have distinct NUE in our previous studies (Supplementary Information S1). The NUE of T29 and T13 was 46.01% and 15.81%, respectively. Cuttings of these varieties, 13.5 ± 1.5 cm in length, were collected and rooted in a hydroponic apparatus with water only in a plant growth chamber in May 2015. During the period of ramie culture, the relative humidity was 60%, temperature was 25 °C, and the photoperiod was 16 h/8 h (light/dark). On the seventh day, ramie plants were divided into two groups and provisioned with 15 L modified Hoagland solution [24] (Table 1). Thirty plants of each variety cultured with 10 mmol·L^−1^ N formed the control group (T29_C and T13_C), and others cultured with 0 mmol·L^−1^ N formed the treatment group (T29_T and T13_T).

Samples from roots, stems, and leaves were collected after 0.5, 1, 3, 5, and 7 days of culturing. Each sample was derived from three plants and two biological replicates. All samples of all treatments were mixed equally and sequenced to construct the transcriptome library. Subsequently, all samples of each treatment were mixed for gene expression analysis. Both treatments of each genotype were replicated twice for Illumina sequencing and DGE analysis. After 45 days of N treatment, shoots and roots were harvested separately for biomass testing.

### 2.2. RNA Isolation and Library Preparation for DGE Sequencing

The total RNA was extracted using an E.Z.N.A.^®^ Plant RNA Kit (OMEGA Bio-Tek, Norcross, GA, USA) according to the manufacturer’s protocol. The RNA-seq and assembly were performed at Novogene Bioinformatics Technology CO., LTD., Beijing, China (http://www.novogene.cn) using the Illumina platform. Briefly, 3 μg total RNA per sample was used and treated with DNase I. The RNA quality and integrity were detected by the Agilent Bioanalyzer 2100 system. RNA-seq libraries were constructed using NEBNext^®^ Ultra™ RNA Library Prep Kit for Illumina^®^ (NEB, Ipswich, MA, USA) according to the manufacturer’s protocol. Library quality was assessed using an Agilent Bioanalyzer 2100 (Agilent Technologies, Santa Clara, CA, USA). Whole RNA sequencing datasets were submitted to NCBI.

### 2.3. Quality Control and Quantification of Gene Expression Levels

Raw reads of FASTQ format were first processed through in-house perl scripts. In this step, clean reads were obtained by removing reads containing adaptors, ploy-N residues, and low-quality reads from the raw data. At the same time, Q20, Q30, GC content, and sequence duplication level of clean data were calculated. All downstream analyses were based on clean, high-quality reads.

Gene expression levels were estimated using RNA-seq by RSEM (RNA-Seq by Expectation Maximization) [25]; clean data of each sample were mapped back onto the assembled transcriptome and a read count for each gene was obtained from the mapping results. Transcriptome assembly was accomplished based on the left.fq and right.fq using Trinity [26] with min_kmer_cov set to 2, and all other parameters at default settings.

### 2.4. Gene Annotation

Gene function was annotated based on the following databases: NCBI Non-redundant Protein Sequences (NR); Swiss-Prot; Gene Ontology (GO); NCBI Nucleotide Sequences (NT); Clusters of Orthologous Groups of proteins (COGs); Protein family (Pfam); and the KEGG Ortholog (KO).

### 2.5. Differential Expression Analysis

The differentially expressed genes of the two treatments were analyzed using the DESeq package in R 1.10.1 (R Foundation for Statistical Computing, Vienna, Austria). DESeq performs statistical analyses to determine differential expression in DGE data using a model based on the negative binomial distribution. The resulting *p* values were adjusted using the Benjamini–Hochberg procedure [27] to control the false discovery rate. Genes with an adjusted *p* value < 0.05 were considered differentially expressed.

### 2.6. Pathway Enrichment Analysis of DEGs

Gene ontology enrichment analysis of differentially expressed genes (DEGs) was done using the GOseq packages in R based on Wallenius’ noncentral hypergeometric distribution [28], which adjusts for gene length bias in DEGs.

KEGG is a database resource for understanding high-level functions and utilities in biological systems [29]. In this study, KOBAS [30] software was used to test the statistical enrichment of differentially expressed genes in KEGG pathways.

### 2.7. Quantitative Real-Time PCR (qRT-PCR) Analysis

Gene quantification was performed using a two-step reaction process: reverse transcription (RT) and PCR. Each RT reaction consisted of 0.5 μg RNA, 2 μL of PrimerScript Buffer, 0.5 μL of oligo dT, 0.5 μL of random hexamers, and 0.5 μL of PrimerScript RT Enzyme Mix I (TaKaRa BioInc., Shiga, Japan) in a total volume of 10 μL. Reactions were performed in an Applied Biosystems^®^ GeneAmp^®^ PCR System 9700 (Thermo Fisher Scientific Inc., Waltham, MA, USA) for 15 min at 37 °C, followed by heat inactivation of RT for 5 s at 85 °C. The 10 μL RT reaction mix was then diluted (1:10) in nuclease-free water and stored at −20 °C.

Real-time PCR was performed using a LightCycler^®^ 480 Real-Time PCR Instrument (Roche, Swiss) with a 10 μL PCR reaction mixture that included 1 μL of cDNA, 5 μL of 2 × LightCycler^®^ 480 SYBR Green I Master (Roche, Swiss), 0.2 μL of forward primer, 0.2 μL of reverse primer, and 3.6 μL of nuclease-free water. Reactions were incubated in a 384 well optical plate (Roche, Swiss) at 95 °C for 10 min, followed by 40 cycles at 95 °C for 10 s, and 60 °C for 30 s. Each sample was run in triplicate. At the end of the PCR cycles, a melting-curve analysis was performed to validate the expected PCR products. Primer sequences were designed in the laboratory and synthesized based on mRNA sequences obtained from the NCBI database.

Messenger RNA expression levels were normalized for 15 genes and were calculated using the 2^−ΔΔCt^ method [31]. Briefly, melting-curve analysis of the amplified products was performed at the end of each PCR run to confirm that only one PCR product was amplified and detected. The Ct values of target genes were thus normalized with the 18S rRNA reference gene, while a mathematical model was used to determine the relative expression ratio. The real-time PCR-primers for validated gene expression in the results of DGEs and housekeeping genes are shown in the Supplementary Information S2.

## 3. Results

### 3.1. The Performance of Ramie Genotypes under Different Nitrogen Treatments

**Figure 1:**
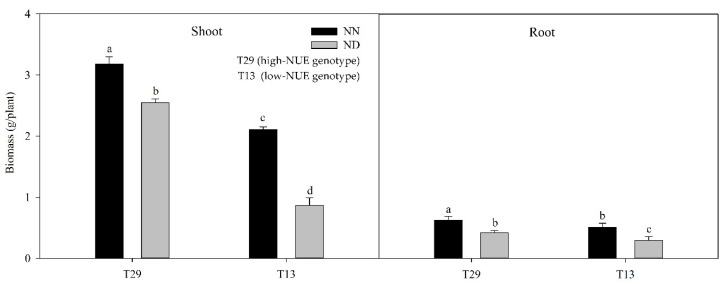
Plant growth performance of ramie genotypes under nitrogen deficiency. ND: N-deficient conditions, NN: normal-N conditions. The error bar represents the standard error. The different letters in the chart indicate significant differences at *p* < 0.05 between the genotypes according to SNK test.

Both the genotypes showed a remarkable decrease in shoot and root biomass after 45 days of treatment under N-deficit stress (Figure 1). Thus, nitrogen deficiency inhibited the growth of ramie regardless of the NUE performance. The shoot biomass of T29 and T13 was decreased by 20% and 59%, respectively. A similar pattern was observed in root biomass. Therefore, we hypothesized that the low-NUE ramie genotype were more sensitive to N deficiency.

### 3.2. De Novo Transcriptome Sequencing in High-NUE and Low-NUE Ramie Genotypes

Mixed samples of four control plants (T29_C1, T29_C2, T13_C1, and T13_C2) and four N-deficit-treated plants (T29_T1, T29_T2, T13_T1, and T13_T2) were used to construct libraries of total RNA. A total of 55.1 million clean sequence reads (96.58% of all raw reads) were obtained at an error rate below 1%. Clean reads were spliced into transcripts using Trinity software, resulting in a total of 80,493 transcripts with an average length of 919 bp. From these transcripts, 61,424 non-redundant unigenes were yielded. Seven public databases were used for validation and annotation of the assembled unigenes. The results indicated that 50.3%, 24.0%, 19.3%, 38.3%, 37.4%, 39.0%, and 23.2% of the unigenes showed similarity to known proteins in the NR, NT, KO, Swiss-Prot, PFAM, GO, and KOG databases, respectively (Table 2). Together, 34,251 (55.8%) unigenes showed similarity to known proteins in at least one of these seven databases.

### 3.3. Global Analysis of Differential Gene Expression

**Figure 2:**
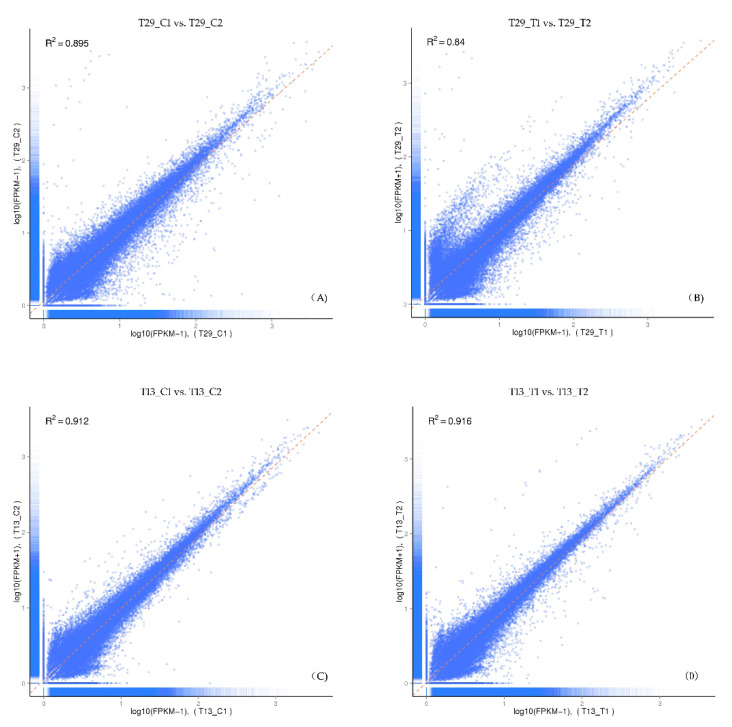
The correlation between the expression levels for each gene for the two biological replicates. Abscissa indicates log10(RPKM + 1) of Biological Replicate 1, Ordinate indicates log10(RPKM + 1) of Biological Replicate 2. T29 is the high-NUE genotype and T13 is the low-NUE genotype. Subfigures (**A**), (**B**), (**C**) and (**D**) show the correlation between the two biological replicates of T29_C (high-NUE genotype T29 under normal N treatment), T29_T (T29 under N-deficit treatment), T13_C (low-NUE genotype T13 under normal N treatment) and T13_T (T13 under N-deficit treatment), respectively.

All sequencing reads were realigned to the unigenes to determine the expression levels of the 61,424 unigenes assembled *de novo*. In total, the DGE libraries generated between 10.1 and 12.2 million raw reads. After removing insignificant reads, the total number of clean reads ranged from 9.93 to 11.89 million. Clean reads per library were mapped to the reference transcriptome database using RSEM software, and represented between 88.31% and 90.20% of the overall total. The FPKM values of all unigenes were compared between replicates. Significant correlations between replicates were observed, which suggested that the abundances of these unigene transcripts in the replicate libraries were similar (Figure 2).

**Figure 3:**
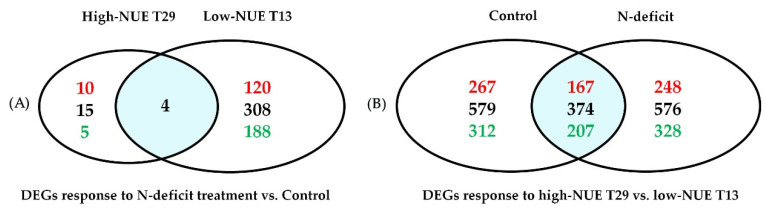
Venn diagram of DEGs in different treatments. The sum of the numbers in each large circle represents the total number of differentially expressed genes between combinations; the overlapping part of the circles colored with cyan represents common DEGs between combinations. Black numbers represent the total DEG number. (**A**) DEGs responding to N deficit compared to control, in which red numbers represent upregulated DEGs and green numbers represent downregulated DEGs. (**B**) DEGs responding to different genotypes, in which red numbers represent DEG numbers in high-NUE T29 and green numbers represent DEG numbers in low-NUE T13.

The numbers of DEGs that were up- and downregulated were determined in the comparisons of the two treatments and genotypes (19, 312, 953, and 950 for all; Figure 3). Among these four comparisons, 1699 unigenes were identified, comprising 1265 unique NR database matches. These genes had an average fold-change of 22.35 (range: 1.49–4465.36) for 739 upregulated and −30.72 (range: −1.4–−7426.18) for 960 downregulated unigenes (Supplementary Information S3). The most highly upregulated gene was a hypothetical protein, POPTR_0008s16330g, belonging to the PHD finger protein family (c57966_g1), which are regarded as key factors in chromatin and transcription regulation [32]. The most downregulated gene was UPL1, a zinc finger protein with E3 ubiquitin ligase activity (c33163_g1). The classifications of the 1265 unigenes included kinase (57 unigenes), transcription factor (31 unigenes), cytochrome P450 (30 unigenes), photosystem (30 unigenes), disease resistance protein (27 unigenes), UDP-glycosyltransferase (14 unigenes), glutathione *S*-transferase (12 unigenes), and GDSL esterase and lipase (10 unigenes), among others.

### 3.4. Genotype-Specific Gene Expression within Treatments

The two genotypes differed significantly in gene expression under the normal-N condition across 953 genes (434 higher in T29 (mean fold-change: 26.65); and 519 higher in T13 (mean fold-change: −53.69)) There were 30 GO categories between T29_C and T13_C related to catalytic activity (351 unigenes) and binding-related activity (130 unigenes) (Supplementary Information S3 and S4). The main significantly enriched categories were lysosome, glutathione metabolism (seven unigenes), retinol metabolism (six unigenes), metabolism of xenobiotics by cytochrome P450 (six unigenes), phenylpropanoid biosynthesis (six unigenes), and carbohydrate digestion and absorption (three unigenes). Under N-deficit conditions, high-NUE genotype T29 and low-NUE genotype T13 differed significantly in gene expression across 950 genes (415 higher in T29 (mean fold-change: 27.63); and 535 higher in T13 (mean fold-change: −25.11)) of these genes, 374 were commonly and differentially expressed between T29_C and T13_C, and T29_T and T13_T. There were 24 GO categories between T29_T and T13_T related to single organism metabolic process (207 unigenes) and oxidoreductase activity (117 unigenes). The main significantly enriched categories were carbon metabolism (20 unigenes), biosynthesis of amino acids (16 unigenes), phenylpropanoid biosynthesis (12 unigenes), and phenylalanine metabolism (9 unigenes).

### 3.5. Variety-Specific Responses to N-Deficit Conditions

Low-NUE genotype T13 (121 upregulated and 191 downregulated genes) showed exceedingly different transcriptome response to N deficit than high-NUE genotype T29 (13 upregulated and 6 downregulated genes). In T13, 308 genes reacted significantly to N deficit that did not in T29.

Not all 308 genes showed the same direction of change in the two varieties. A downregulated gene was abandoned due to lack of significant change in T29. A total of 121 upregulated genes showed a greater increase, and 187 downregulated genes showed a greater decrease in T13 (χ^2^ test; *p* < 0.01).

**Figure 4:**
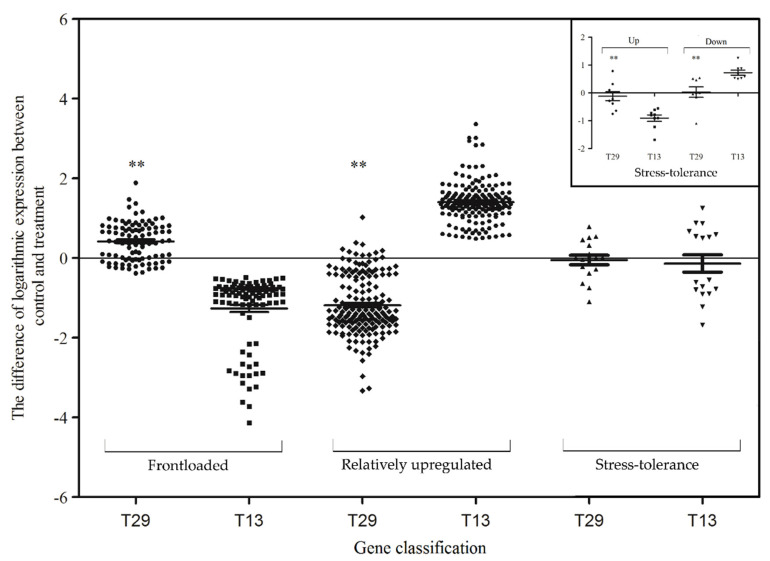
Scatter plot of expression of genes between high-NUE T29 and low-NUE T13, unique to the T13_T vs. T13_C. The difference of logarithmic expression between control and treatment is shown on the vertical axis ln(T29_C/T29_T) and ln(T13_C/T13_T); the types of differentially expression genes are shown on the horizontal axis. The small figure shows the up- and downregulated details of stress-tolerance genes in T13. ** indicates significant difference between the two genotypes at *p* < 0.01, vertical error bars represent mean ± SD.

In total, 94 of 120 genes in the high-NUE genotype T29_T, displayed reduced upregulation compared with higher expression in T29_C and T13_C (i.e., each gene’s ratio in T29_C and T13_C expression levels) and lower expression levels in T29_T and T13_T, i.e., each gene’s ratio in T29_T and T13_T fold-change values in the N-deficit treatment (Figure 4). The relationship showed that genes reacted less sensitively during N-deficit conditions in T29, and may have exhibited a higher level of gene expression before the onset of N-deficit. Another potential explanation is that many upregulated genes in T13 were already expressed at higher levels in T29, accounting for our results.

**Figure 5:**
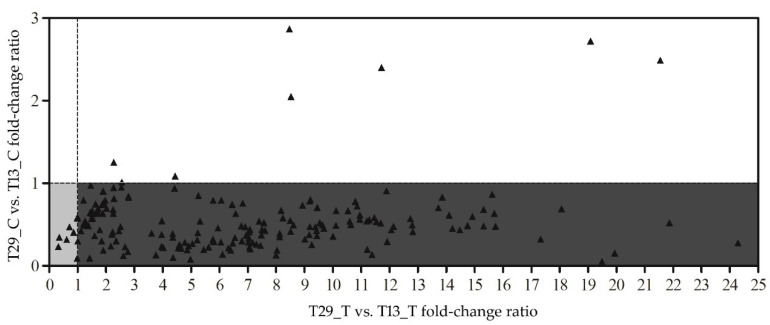
Scatter plot comparing the relative ratio of treatment-to-control fold-changes in expression between high-NUE T29 and low-NUE T13 across the 187 downregulated differently expressed genes unique to the T13_T vs. T13_C. y < 1 T29 control showed lower expression relative to T13 control, x > 1 T29 treatment showed higher expression relative to T13 treatment. Numbers greater than 25 are not shown. The gray portion of the graph represents the stress-tolerance genes and the dark portion represents relatively upregulated genes.

Nine of 120 upregulated genes and 8 of 187 downregulated genes (Figure 5) responded less sensitively to N-deficit in T29 compared with T13_T, and showed equal or even lower expression in the T29_C compared with T13_C. The results showed that the high-NUE genotype T29 had a reduced reaction during N deficiency.

A total of 94 genes had higher constitutive expression levels under the control treatment but had a lower response to N deficiency in the high-NUE T29. These genes included GDSL esterase and lipase, zipper protein, NADH dehydrogenase, and UDP-glycosyltransferase. A total of 17 genes had lower constitutive expression levels and a lower reactivity to N deficiency in T29, including auxin-induced protein, 1-aminocyclopropane-1-carboxylate oxidase-1-like protein, and inter-alpha-trypsin inhibitor heavy chain H3. There were 170 genes that were relatively upregulated in T29 but downregulated in T13, including wall-associated receptor kinase, serine carboxypeptidase, homeobox-leucine zipper protein, and ferulate 5-hydroxylase. These genes represent potential candidates contributing to N-deficit-stress resistance. Overall, there were three markedly different expression patterns of these genes. Transcripts of these three gene categories were annotated with 15 GO classification terms, including single organism metabolic process (85 unigenes), oxidoreduction (63 unigenes), binding-related (19 unigenes), and dioxygenase activity (14 unigenes).

**Figure 6:**
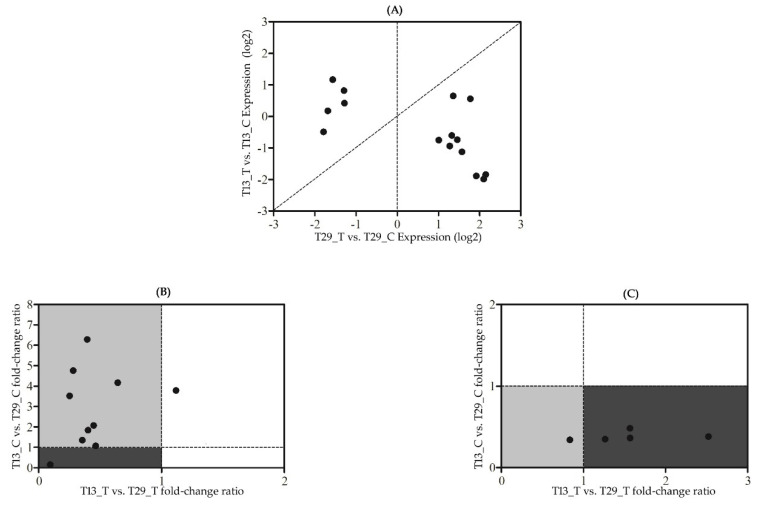
Scatter plot of expression between low-NUE T13 and high-NUE T29 across the 15 differently expressed genes unique to T29_T vs. T29_C. (**A**) Scatter plot of the log2 fold-changes in expression of 15 genes that were unique to T29_T vs. T29_C in response to N-deficit stress in T29 and T13. (**B**) Comparison of the relative ratio of treatment-to-control fold-changes in expression of the 10 upregulated DEGs unique to T29_T vs. T29_C between T29 and T13. (**C**) Comparison of the relative ratio of treatment-to-control fold-changes in expression of the five downregulated DEGs unique to T29_T vs. T29_C between T29 and T13. Each filled dot represents an individual gene; the dotted-line is a 1:1 line; the gray and dark portions of the (**B**) graph represent the frontloaded and stress-tolerance genes, respectively; the gray and dark portions of the (**C**) graph represent stress-tolerance or relatively upregulated genes, respectively.

In contrast, eight genes with reduced upregulation in T13_T showed higher expression in T13_C and T29_C but lower expression in T13_T and T29_T (Figure 6). Two genes showed equal or lower expression in T13_C compared with T29_C, which showed a reduced reaction during N deficiency in the low-NUE genotype. Four genes were downregulated in T29 that responded as relatively upregulated in N-deficient stress in T13. Five of eight transcripts were annotated with one GO classification term, including oxidoreductase activity, acting on paired donors, with incorporation or reduction of molecular oxygen. DEGs were significantly enriched in alpha-linolenic acid metabolism.

There were four genes that displayed opposite expression patterns under N deficiency in T29 and T13. In T29, three genes were upregulated and one gene was downregulated, and T13 displayed the opposite pattern. The role of these genes in N-deficiency stress is unclear.

### 3.6. Quantitative Real-Time PCR

To validate the reliability of Illumina sequencing technology, 15 genes were randomly selected from both of the genotypes under N-deficit treatment for quantitative RT-PCR assays. The results showed that all 15 of these genes had different expression levels in the treatment and control, and the trend of expression changes based on qRT-PCR was the same as that detected by DGE analysis (Supplementary Information S5).

## 4. Discussion

### 4.1. Feasibility of Gene Expression Pattern in Ramie NUE

Among the essential plant nutrients, N plays the most important role in improving the agricultural production of ramie [33]. Hence, it is important to maximize nitrogen-use efficiency. As nitrogen-saving cultivation becomes a trend, such as ISSM (Integrated Soil-crop System Management) [34], strategies combining high yield and low nitrogen rate together should be applied in ramie farming. In the present study, significant differences were found in whole plant biomass, above-ground biomass, and shoot/root ratio between the two varieties under both normal and N-deficit conditions. Additionally, the genome-wide expression profile of molecular response to N deficiency in different ramie varieties was characterized for the first time. A total of 170 N-stress-responsive genes were identified. The results suggest that the NUE of ramie could be improved significantly by genetic strategies, and ramie cultivation could be operated more economically by using varieties with higher NUE under a lower nitrogen rate.

### 4.2. Functional Classification of N-Stress-Responsive Genes in Ramie

A number of studies have found that the basic ramie stress response involves a wide array of molecular processes which cause differences in physiological resilience [35,36,37]. Similarly, in the present study, genes related to numerous physiological functions were identified to be responsive to nitrogen deficiency. However, none of the previous studies involved varieties with contrast traits. Numerous genes showed altered expression levels under N-deficit conditions, and many of the genes that showed striking different responses to N deficiency in low-NUE genotypes showed reduced response in the high-NUE genotypes (Supplementary Information S6). That is, we found that low-NUE and high-NUE ramies showed different patterns of gene expression under N deficiency. Based on the transcriptome and the differential expression pattern, we categorized the potential key genes related to nitrogen utilization of ramie: (1) frontloaded genes, which already exhibiting a higher gene expression level in high-NUE genotypes compared with low-NUE genotypes under control conditions, but showed a reduced response to N-deficit in a single-genotype-experiment; (2) relatively upregulated genes, which displayed relatively high expression levels under N-deficit stress in high-NUE genotypes, but were downregulated in low-NUE genotypes; and (3) stress-tolerance genes, which showed lower expression levels and were not sensitive to N-deficiency stress in high-NUE genotypes compared to low-NUE genotypes. These gene categories may be useful for exploring the potential mechanisms of high NUE, and to explain why high-NUE genotypes can grow and yield well under N-deficiency stress. We discuss the possible mechanisms of these genes on nitrogen-utilization regulation in the following paragraphs.

### 4.3. Frontloaded Genes

Frontloaded genes can be defined as the genes already upregulated under control conditions in tolerant populations before responding to stress. Barshis et al. [38] proposed that constitutive preloading enables an individual coral (*Acropora hyacinthus*) to maintain physiological resilience during frequently encountered environmental stresses, an idea that has strong parallels in model systems such as yeast (*Saccharomyces cerevisiae*). A total 94 potentially frontloaded genes were found in T29 in our study. Among these genes identified, some might be critical in response to N deficiency, such as GDSL esterase and lipase [39,40,41], gibberellin 20-oxidase and 3-beta-dioxygenase [42,43,44,45], UDP-glycosyltransferase [46,47], and ω-6 fatty acid desaturase [32,48]. These results suggest that these high-NUE ramie genotypes with more physiological resilience follow a similar pattern of gene preloading.

Systemic acquired resistance refers to a distinct signal transduction pathway implicated in the ability of plants to defend themselves against biotic and abiotic stresses [49]. Biotic and abiotic stresses prime subsequent changes in tolerance in many plants. In eggplant, seedlings grew well under cold stress through enhanced antioxidant enzyme activity and related gene expression if they received a salicylic acid pre-treatment at a concentration of 0.3% [50]. In maize and cucumber, a pre-treatment with H_2_O_2_ and paraquat reduced salt-induced oxidative damage by increasing the antioxidative mechanisms [51,52]. These results are similar to the increased adaptability to N deficit seen in the high-NUE genotype T29. Frontloaded genes may reduce stress through faster protein mobilization and produce results similar to those acquired with pre-treatments.

### 4.4. Relatively Upregulated Genes

Numerous genes showed relatively high expression levels under N deficiency in the high-NUE genotype and were downregulated in the low-NUE genotype, including wall-associated receptor kinase (WAK), serine carboxypeptidase (SCP), and homeobox-leucine zipper protein (HD-Zip). Previous studies have suggested that some WAK members play an important role in responses to aluminum, cell elongation, and plant development [53,54,55]. Serine carboxypeptidases comprise a large family of protein-hydrolyzing enzymes that play roles in multiple cellular processes. *OsBISCPL1* and *GS5* encode a putative SCP, and *OsBISCPL1*-overexpressing plants showed an increased tolerance to oxidative stress and upregulated expression of oxidative-stress-related genes. Higher expression levels of *GS5* can increase grain size and yield in rice [56,57]. Homeodomain-leucine zipper proteins are transcription factors unique to plants. These proteins are generally involved in responses related to abiotic stress, abscisic acid (ABA), blue light, de-etiolation, and embryogenesis [58]. WAK, SCP, and HD-Zip are involved in reducing damage due to various stresses. A number of differentially expressed genes followed a similar pattern of relative upregulation in the high-NUE genotype. We hypothesized that these genes respond to nitrogen stress, polarize expression among varieties, and then lead to differentiation in production performance.

### 4.5. Stress-Tolerance Genes

Some genes responded less sensitively to N deficiency in high-NUE genotypes, and showed equal or even lower level of gene expression in T29_C compared with T13_C. In our dataset, 5.52% of the 308 reduced-reaction genes fell into this category, of which, 9 were upregulated and 8 were downregulated. In these stress-tolerance genes, one was a match to the inter-alpha-trypsin inhibitor heavy chain H3 precursor (ITIH3; c32816_g1 and c34225_g1; Supplementary Information S7), and the other was a BLAST match to 1-aminocyclopropane-1-carboxylate oxidase-1-like protein (ACC; c21936_g1 and c25703_g1). ACC synthase is a rate-limiting oxidase in the regulation of the ethylene biosynthesis. As a plant hormone, ethylene plays a significant role in plant maturation and senescence [59,60]. Saving energy and transferring substances for essential life activities are important to survival in adverse environments [61]. The present study showed that reduced expression changes in T29 might delay senescence, which would enable the plants to be resilient than T13 under N-deficiency stress.

## 5. Conclusions

Ramie plants respond to the environment in a complex fashion, and the magnitude of changes in gene expression was different between genotypes under N-deficiency stress. Many studies on the effect of low-N conditions on gene expression in one or two genotypes have been reported. However, our results went a step further to reveal the resilience-regulation mechanism in ramie by using two genotypes contrasting in NUE. The study detected many DEGs responding to nitrogen deficit, and we categorized these genes into three main groups based on the transcriptome and their expression patterns. A total 94 genes showed a higher expression level in high-NUE genotype T29 before exposure to N-deficient conditions compared to the low-NUE genotype T13; these are defined as frontloaded genes, which are important in reacting to N-deficiency stress in high-NUE genotypes. A total of 17 genes responded less sensitively to N deficiency in the high-NUE genotype T29, and showed equal or even low expression levels compared to the low-NUE genotype T13 under normal N conditions. Defined as stress-tolerance genes, these might enable the plant to have a more resilient adaptability to N deficiency. A total of 170 genes responded with relatively high expression levels under N deficiency in the high-NUE genotype, and were downregulated in the low-NUE genotype; defined as relatively upregulated genes, these were major drivers for NUE differentiation in ramie. There three categories of genes express patterns were used to construct a multi-dimensional molecular mechanism of ramie in N-stress response. These concepts could provide further insight into the mechanism linking these three gene classes and the enhanced resilience of high-NUE genotypes. We identified only eight frontloaded genes, two stress-tolerance genes, and four relatively upregulated genes in the low-NUE genotype. This result explains why high-NUE genotypes have increased yield and growth under N-deficiency stress than low-NUE genotypes. In addition, the three gene categories presented a hypothesis regarding the way ramie responds to N-deficiency stress, which might serve to explain other differences in plants under biotic and abiotic stresses. Different patterns of gene expression under different abiotic conditions (N deficiency) should be taken into consideration for ramie molecular breeding.

## Figures and Tables

**Table 1:** Components of macro- and micro-elements in nutrient solution.

Salts		Concentration (mg·L^−1^)	
		N0	N10
Macro-elements	Ca(NO_3_)_2_ 4(H_2_O)	-	944.6
	KNO_3_	-	202.2
	KCl	-	223.65
	K_2_SO_4_	435.65	-
	KH_2_PO_4_	136.07	136.07
	MgSO_4_ 7H_2_O	492.96	492.96
	CaCl2	554.9	110.98
Micro-elements	H_3_BO_3_	2.86	2.86
	MnSO_4_·H_2_O	1.55	1.55
	ZnSO_4_·7H_2_O	0.22	0.22
	CuSO_4_·5H_2_O	0.08	0.08
	H_2_MoO_4_·4H_2_O	0.09	0.09
	FeNa·EDTA	13.00	13.00

**Table 2:** Functional annotation of the ramie transcriptome in the seven public databases searched.

Database	Number of Unigenes	Matching Proportion
NCBI Non-redundant Protein Sequences (NR)	30,919	50.3%
NCBI Nucleotide Sequences (NT)	14,730	24.0%
KEGG Ortholog (KO)	11,856	19.3%
Swiss-Prot	23,502	38.3%
Protein family (Pfam)	22,973	37.4%
Gene Ontology (GO)	23,968	39.0%
Clusters of Orthologous Groups of proteins (COGs)	14,230	23.2%
Total	34,251	55.8%

## Supplementary Materials

The following are available online at https://www.mdpi.com/2223-7747/9/6/767/s1, S1 NUE of ramie genotypes used in the screening experiment. S2 Real-time PCR-primers for validated gene expression in results of DGEs and housekeeping genes. S3 Results for the 1699 differentially expressed genes (DEGs) between control and treatments. S4 Summary of the part differentially expressed genes grouped by functional category. S5 The DGEs validated by qRT-PCR. S6 Category of DGEs in T29 showed different responding characteristics under nitrogen deficit condition. S7 308 unigenes that were unique to the T13 DEG set.
